# FOXQ1, a Novel Target of the Wnt Pathway and a New Marker for Activation of Wnt Signaling in Solid Tumors

**DOI:** 10.1371/journal.pone.0060051

**Published:** 2013-03-26

**Authors:** Jon Christensen, Susanne Bentz, Thierry Sengstag, V. Prasad Shastri, Pascale Anderle

**Affiliations:** 1 Institute of Macro Molecular Chemistry, Albert-Ludwigs-University of Freiburg, Freiburg, Germany; 2 BIOSS Centre for Biological Signalling Studies, Albert-Ludwigs-University of Freiburg, Freiburg, Germany; 3 Institute of Biochemistry and Molecular Medicine, University of Bern, Swiss National Centre of Competence in Research TransCure, University of Berne, Berne, Switzerland; 4 OSC-Omics Science Center, RIKEN Yokohama Institute, Yokohama, Japan; 5 Swiss National Centre of Competence in Research Molecular Oncology, Lausanne, Switzerland; 6 Swiss Institute for Experimental Cancer Research, Ecole Polytechnique Fédérale de Lausanne, School of Life Sciences, Lausanne, Switzerland; 7 Swiss Institute of Bioinformatics, Lausanne, Switzerland; University of Alabama at Birmingham, United States of America

## Abstract

**Background:**

The forkhead box transcription factor FOXQ1 has been shown to be upregulated in colorectal cancer (CRC) and metastatic breast cancer and involved in tumor development, epithelial-mesenchymal transition and chemoresistance. Yet, its transcriptional regulation is still unknown.

**Methods:**

FOXQ1 mRNA and protein expression were analysed in a panel of CRC cell lines, and laser micro-dissected human biopsy samples by qRT-PCR, microarray GeneChip® U133 Plus 2.0 and western blots. FOXQ1 regulation was assayed by chromatin immunoprecipitation and luciferase reporter assays.

**Results:**

FOXQ1 was robustly induced in CRC compared to other tumors, but had no predictive value with regards to grade, metastasis and survival in CRC. Prototype-based gene coexpression and gene set enrichment analysis showed a significant association between FOXQ1 and the Wnt pathway in tumors and cancer cell lines from different tissues. *In vitro* experiments confirmed, on a molecular level, FOXQ1 as a direct Wnt target. Analysis of known Wnt targets identified FOXQ1 as the most suitable marker for canonical Wnt activation across a wide panel of cell lines derived from different tissues.

**Conclusions:**

Our data show that FOXQ1 is one of the most over-expressed genes in CRC and a direct target of the canonical Wnt pathway. It is a potential new marker for detection of early CRC and Wnt activation in tumors of different origins.

## Introduction

The canonical Wnt pathway plays an important role in embryogenesis and cancer development [1]. The hallmark of Wnt activation is the nuclear accumulation of its main effecter molecule β-catenin. In the nucleus, β-catenin is a component of the transcriptional activation complex that includes members of the TCF/LEF family of DNA binding proteins [2], [3].

Wnt has been shown to contribute to cancer progression in a variety of tumors [4]. Most evident is the role of Wnt signalling in colorectal cancer (CRC) [5]. Here mutational activation of the Wnt pathway is considered the initiating event and is required for the formation of tumors [5]. Familial adenomatous polyposis is a hereditary condition where the APC gene is mutated, which directly leads to increased Wnt activity. Patients with these mutations develop CRC at an early age if not treated [6]. About 85% of all sporadic and hereditary colorectal tumors show loss of APC function [2]. Among the 15% of CRCs that retain wildtype APC, point mutations were found in ß-catenin which change any of the four serine/threonine residues in its N terminus, the putative targets of GSK-3 [5]. Recent findings by the Cancer Genomic Atlas Network suggest that 93% of CRCs have an altered Wnt signaling pathway [7]. Sixteen different genes involved in Wnt signaling were found to be altered with APC and ß-catenin being the most frequent [7]. In breast, lung and prostate cancer Wnt activity has been linked to an increased risk of metastasis, cancer stem cell properties and to induce epithelial to mesenchymal transition (EMT) [8]–[11].

Identified in 1998 in mouse the transcription factor forkhead box Q1 (Foxq1) was shown to be responsible for a defect in hair follicle development in the satin mouse strain [12]–[14]. Through analysis of knockout mice Foxq1 has been shown to play a role in gastric acid secretion and mucin gene expression [15], [16]. Recently, it was shown that FOXQ1 increased tumorigenesis of colon and breast cancer cells. FOXQ1 was upregulated in CRC and in metastatic breast cancer cell lines, and could protect lung, breast and colon cancer cell lines from chemotherapy induced apoptosis and regulate EMT in CRC and breast cancer cell lines [17]–[19]. The mechanisms regulating FOXQ1 expression in CRC and metastatic breast cancer cell lines have not been examined. Reports have indicated that FOXQ1 is a downstream mediator of Hoxa1 and TGFß signalling pathways, but no molecular evidence exists for a direct regulation [19], [20], nor has in-depth analysis been done of genes and pathways associated with FOXQ1. Through the use of public available expression data sets we have examined the expression of FOXQ1 in solid tumors and cancer cell lines. We show that FOXQ1 is one of most upregulated genes in CRC, however, the level of expression was not associated with metastasis, stage or grade. For the first time we demonstrate that FOXQ1 is a direct Wnt target and the expression level correlates with the overall expression of Wnt genes in cancer biopsies and cell lines, thus, demonstrating its potential as a marker for Wnt activation.

## Materials and Methods

### Cell lines

The epithelial cell lines SW480, SW620 and COLO320 were cultured in RPMI 1640 containing 10% fetal calf serum (FCS), MDA-MB-231, HT29, LS174T and HEK293T in Dulbecco's modified Eagle essential medium (DMEM) Glutamax containing 10% FCS, Caco-2 were cultured in DMEM Glutamax, 10% FCS supplemented with 1% NEAA, T84 in RPMI 1640 containing 10% FCS and 2 mM glutamine, and HCT116 (generously provided by Dr. M. Aguet at the ISREC/EPFL, Switzerland) in McCoýs medium containing 10% FCS [21]. All of the cell lines were obtained from LGC Standards (LGC Standards, France) if not stated otherwise. Primary fibroblasts were cultured in DMEM Glutamax supplemented with 10% FCS, 5 mL penicillin/streptomycin and 0.5 mL gentamycin [21]. Caco-2 CDX2 knock-down cells were a generous gift from Drs. Felsani and Natoli (CNR, Italy). All cell lines were cultured at 37°C and 5% CO_2_.

### Reporter plasmids

The human FOXQ1 promoter region was amplified from MDA-MB-231 human breast cancer cells. The resulting 2.5 kb wildtype amplicon was cloned into the pGL3-Basic vector (Promega, Germany) producing pGL3-FOXQ1_WT. Mutation of the TCF4 binding site was done by site-directed mutagenesis thus generating (Strategene, Germany) pGL3-FOXQ1_MUT. Information about primers is presented in Table S1.

### Wnt activation and luciferase reporter assay

The Wnt pathway was activated in HEK293 and HCT116 cells via introduction of a constitutively active form of ß-catenin harbouring mutations in the GSK-3 recognition site (pcDNA3-S33Y, Addgene plasmid 19286) [22] or by inhibiting GSK-3 activity by lithium chloride (LiCl, Sigma-Aldrich) treatment [23], [24]. Briefly, cells were treated with LiCl at a final concentration of 20 mM for 24 hours and analysed, or cells were transfected with pcDNA3-S33Y using Lipofectamine 2000 (Invitrogen, Germany) according to manufactures protocol, selected with 600 µg/mL G418 and analysed.

Luciferase reporter assay was carried out according to manufacturer's protocol. Briefly, HEK293 and HCT116 cells were transfected with pRL-TK as control for transfection efficiency (Promega, Germany) and one of the FOXQ1 promoter constructs (pGL3-FOXQ1_WT, pGL3-FOXQ1_Mut or pGL3-Emtpy) using Lipofectamine 2000 (Invitrogen, Germany). The Wnt pathway was activated as described above and cells were analysed for renilla luciferase and firefly luciferase activity 48 hours after transfection by measuring bioluminescence on a Synergy HT (BioTek, Winooski, Vermont USA) using Dual-Glo (Promega, Germany). Data is represented as firefly luciferase activity normalized to renilla luciferase activity.

### Quantitative real-time PCR

Total RNA was extracted using Nucleo-Spin RNA-extraction kit from Machery-Nagel (Oensingen, Switzerland). Reverse transcription of RNA to cDNA was done using M-MLV (Promega, Switzerland). Quantitative real-time PCR (qRT-PCR) was performed using SYBR-Green PCR assay (Applied Biosystems, Switzerland) on ABI 7900 machine (Applied Biosystems, Switzerland), with the following thermal cycling condition: 2 min at 50°C, 10 min at 95°C followed by 40 cycles at 95°C for 15 s and 60°C for 1 min. Primer sequences are listed in Table S1. All biological samples were measured in triplicates and the resulting data were normalized to H3.

### Western blot analysis

For immunoblotting cells were lysed in laemmli buffer and protein concentration was determined using BCA protein assay kit (Pierce, Germany). 30 µg protein was loaded per sample and resolved on a 8% polyacrylamide–SDS gels and transferred onto nitrocellulose membranes, which were immediately blocked with 2% BSA for 1 h. Membranes were then probed with antibodies against FOXQ1 (Sigma, AV33747). GAPDH (Santa Cruz Biotechnology, SC-25778) or Tubulin (Santa Cruz Biotechnology, Santa Cruz, USA, SC-52586) were used a loading control. Blots were developed using peroxidise-conjugated secondary antibodies, and chemiluminescence system (Thermo Scientific, Germany).

### Chromatin immune precipitation

SW480 cells were cross-linked with 1% formaldehyde in PBS for 10 min at room temperature and the fixation was stopped by adding glycine at a final concentration of 0.125 M. Pelleted cells were resuspended and sonicated to produce DNA fragments with a size of <1000 bp (input). Samples were immunoprecipitated overnight at 4°C using 3 μg of ß-catenin antibody (BD Transduction Laboratories, 610154) or negative control antibody (Upstate Millipore, catalogue# 12-371, Switzerland) and protein-A magnetic beads (Upstate, Millipore, catalogue# 16-611, Switzerland). Antibody-chromatin complexes were washed, eluted and crosslinking was reversed in 10% SDS, 1 M NaHCO3, NaCl 5 M for 5 hours at 65°C. Antibodies and beads were separated in EDTA 0.5 M, TrisHCl 1 M pH 6.5, protein kinase K at 45°C for 2 hours and at 95°C for 10 minutes. DNA was recovered and purified. The immunoprecipitated DNA was quantified by qRT-PCR. The amount of immunoprecipitated DNA was calculated in reference to a DNA standard curve and normalized to input and GAPDH control for each experiment. Primer sequences are listed in Table S1.

### Lentiviral production and transduction

SW480 and HEK293T cells were transduced with lentiviral particles carrying short hairpin RNÁs targeting FOXQ1 (shFOXQ1mir) or the Wnt sensitive reporter construct 7TGC (Addgene plasmid 24304) [25]. Lentiviral particles were produced in 293FT cells (Invitrogen, Switzerland), cotransfected with lentiviral vector and packaging vectors. Lipofectamine 2000 (Invitrogen, Switzerland) was used as transfection reagent. 293FT cells were grown in DMEM media supplemented with 10% FCS and non-essential amino acids (Invitrogen, Switzerland). For transfection, 30 µg of DNA was diluted in 1.5 mL Opti-MEM (Invitrogen, Switzerland) with a ratio of 4∶3∶1 of transfer vector (shFOXQ1 or 7TGC), packaging coding vector (pCMV-dR8.74) and envelope coding vector (pMD2.G). 30 µL of Lipofectamine 2000 was diluted in 1.5 mL of Opti-MEM. The two solutions were combined following 5 min incubation at room temperature. The resulting mixture was added to 293FT cells after 1 hour of incubation at room temperature. 16 hours after transfection the media was changed to RPMI with 10% FCS. 40 and 64 hours after transfection of 293FT cells the viral supernatants were collected and centrifuged at 200× g for 5 minutes, and filtered through a sterile 0.45 µm syringe filter (Millipore, Switzerland). The viral particles were pelleted on a 25% sucrose gradient using Optima LE-80K (Beckman Coulter, Switzerland). Concentrated viral particles were added to target cells and transduction efficiency was assayed by FACS (BD FACSCalibur) of the fluorescence transgenes.

### Fluorescence microscopy

HEK293 and HCT116 cells were cultured in ibidi µ-Plate 96 wells (ibidi, Germany). Cells were treated with LiCl or transfected with pcDNA3-S33Y as described previously and live cells were imaged by fluorescence microscopy (Cell Observer, Carl Zeiss, Germany).

### Prediction of transcription factor binding sites

The web base tool Transcription Element Search System (TESS) was used to predict transcription factor binding sites (http://www.cbil.upenn.edu/cgi-bin/tess/tess) [26].

### Data analysis

Robust multi-array averaging (RMA) and quantile normalization were used to quantify gene expression. Significant differences were identified applying a Bayesian approach using the limma package (R 2.12.0, Bioconductor 2.7). A threshold of an adjusted P value ≤0.05 was used to identify significant changes if not indicated otherwise. Prototype-based Gene Coexpression (PGC) was performed as described earlier using the expO data set GSE2109 [27]. Gene set enrichment analysis (GSEA) was carried out according to Subramanian et al. [28] and p values were computed using a bootstrap distribution created by resampling gene sets of the same cardinality. The gene sets were defined as follows: direct Wnt targets: see [29], others: see MiSigDB, http://www.broadinstitute.org/gsea/msigdb/index.jsp), only signatures whose name contained either “WNT” or “CTNNB1” and p values <0.01 were considered. Effects of signatures on overall and recurrence free survival were studied using the Kaplan-Meier survival curves and log rank tests in three different publicly available data sets (GSE12945, GSE14333, GSE17537) using the survival package in the statistical software R version 2.13.0. Patient samples were divided into two groups splitting at the average score. Significant differences of microarray and qRT-PCR data were determined using a Student's t-test, Mann_Whitney U, or Kruskal-Wallis with Bonferroni correction depending on the data structure and the number of groups (p value ≤0.05). In general, genomics data are shown as Box-and-Whisker Plots, i.e. depicting the smallest observation (sample minimum), lower quartile, median, upper quartile, and largest observation (sample maximum). Correlation coefficients of relative expression levels of single direct Wnt targets with mean expression of all targets was performed in the panel of 967 cell lines (GSE36133). The relative expression across cell lines was determined by gene-wise zero-centering of expression values (Details see [21]).

### Publicly available data set

Publicly available data was obtained at http://www.ncbi.nlm.nih.gov/geo/through the accession numbers GSE2109 (expO data), GSE5209, GSE10780, GSE21422, GSE22544, GSE3744, GSE5764, GSE6791, GSE15960, GSE23878, GSE3029, GSE4183, GSE15960, GSE4183, GSE11151, GSE14762, GSE11151, GSE19188, GSE17951, GSE3325, GSE12945, GSE14333 and GSE17537, GSE36133, GSE9452, GSE11223.

## Results

### FOXQ1 is over-expressed in CRCs at all stages and grades

**Figure 1:**
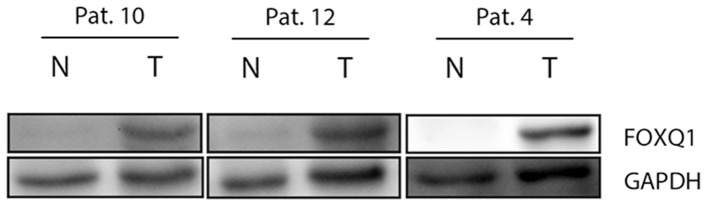
FOXQ1 is induced on protein level in human colon carcinoma as compared to normal tissue. Western blot analysis of FOXQ1 in human CRC biopsy samples from three different patients. GAPDH was used as loading control. T = tumor tissue, N = normal colon tissue.

**Figure 2:**
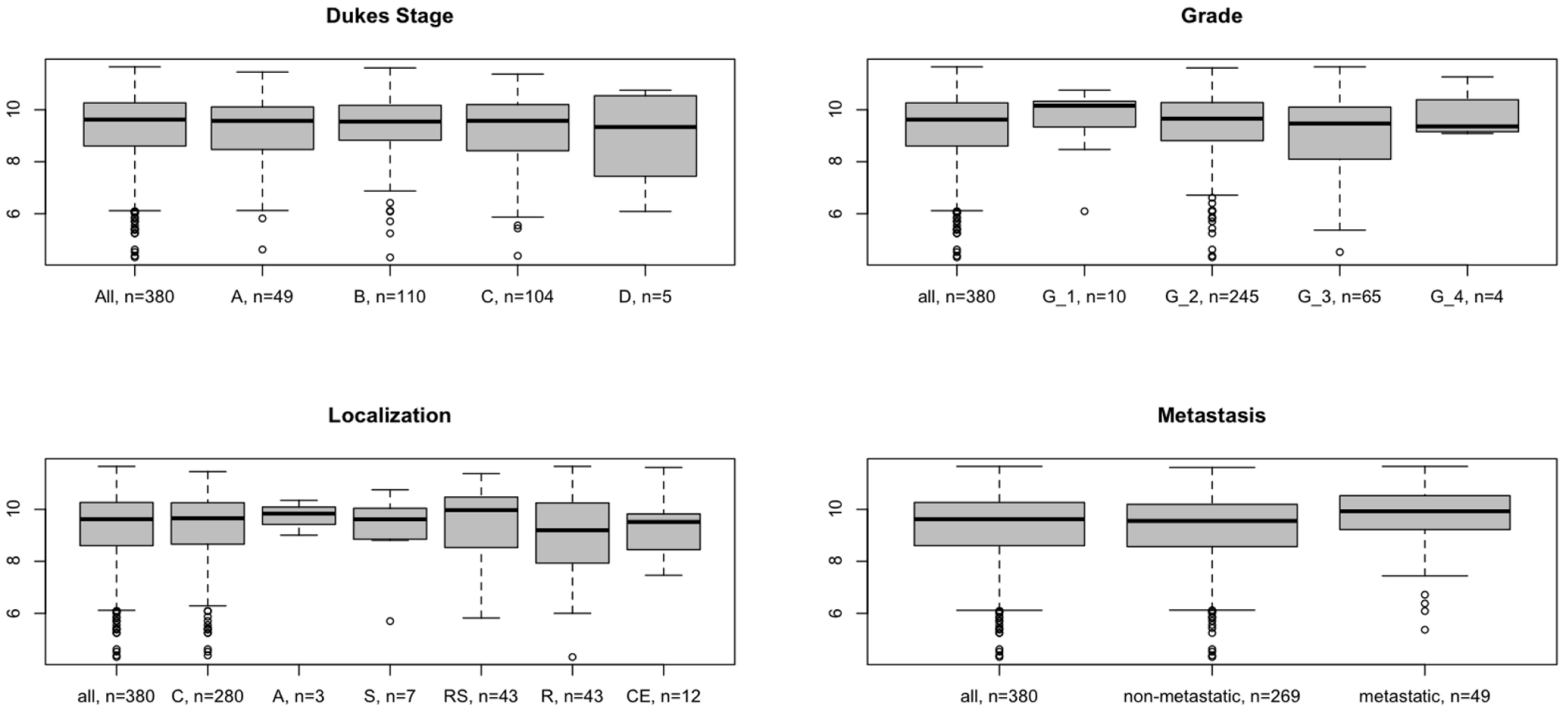
FOXQ1 expression is not influenced by stage, grade and localization of the tumor. Boxplots of FOXQ1 mRNA expression levels (i.e. normalized, log2 transformed fluorescence signals) in the GSE2109 data set. Dukes Stages: stage A, B, C and D; Grades: grades 1, 2, 3 and 4; Localization: C = colon, A = ascending colon, S = sigmoid colon, RS = recto-sigmoid, R = rectum and CE = cecum. Metastasis: non-metastatic = no metastatic site observed, metastatic = metastatic site observed.

The mRNA level of FOXQ1 has been shown to be induced in colon adenoma and carcinoma samples [17], [30]. To verify this, our own and publicly available microarray data set of CRC samples were examined for FOXQ1 expression and showed that FOXQ1 always ranked in the top of all genes being induced in CRC (Table 1). FOXQ1 was also significantly induced on the protein level as compared to normal tissue (Figure 1). Stratifying CRC biopsy samples based on tumor stage, grade, localization and metastatic status revealed no significant differences of FOXQ1 expression level (Figure 2, Figure S1). Overall and recurrence free survival were not changed in CRC patients based on FOXQ1 expression (data not shown). Analysis of expression data from other solid tumors showed that FOXQ1 mRNA level was not significantly induced as compared to normal tissue (Table 1). Overall comparison in different tumor tissues of the expO data, the NCI60 cell line panel and a panel of 967 cell lines revealed that FOXQ1 median expression was highest in CRC compared to all other solid tumors (Figure S2, Figure S3, and Figure S4).

**Table 1:** FOXQ1 expression in tumor samples.

Tissue	Data set	Sample#	Rank	log2 FC	log2 AS	adj. p
Colon	GSE30292	T = 3, N = 3	1	5.55	9.09	0.02
Colon	GSE23878	T = 35, N = 24	1	4.12	7.62	0.00
Colon	GSE15960	T = 6, N = 6	1	6.67	8.59	0.00
Colon	GSE15960	A = 6, N = 6	5	3.33	8.59	0.00
Colon	GSE4183	T = 15, N = 15	34	2.38	9.52	0.00
Breast	GSE5764	T_L = 5, N_L = 10	13013	−0.07	5.63	0.99
Breast	GSE5764	T_D = 5, N_D = 10	13475	−0.11	5.63	0.98
Breast	GSE3744	T_B = 18, N = 7	1655	0.76	5.98	0.10
Breast	GSE3744	T_NB = 20, N = 7	13078	−0.09	5.98	0.89
Breast	GSE3744	T_B = 18, T_NB = 20	709	0.86	5.98	0.01
Breast	GSE21422	T = 5,N = 5	18087	−0.19	5.18	0.77
Breast	GSE22544	T = 14, N = 4	856	1.15	5.04	0.14
Breast	GSE10780	T = 22, N = 25	17432	−0.38	5.69	0.04
Cervix	GSE6791	T = 20, N = 8	15532	−0.33	11.07	0.52
Kidney	GSE11151	T = 26,N = 3	20074	−1.29	8.77	0.34
Kidney	GSE14762	T = 10, N = 11	20249	−1.47	8.66	0.00
Lung	GSE19188	T = 47,N = 40	13409	−0.04	8.28	0.93
Prostate	GSE3325	T = 7, N = 6	20768	−1.71	7.17	0.00
Prostate	GSE17951	T = 23, N = 23	19693	−0.84	10.52	0.03

FOXQ1 belongs to the most overexpressed genes in human colon adenoma and carcinoma as compared to normal tissue. Rank orders, fold changes (FC) and average intensity signals (AS) of FOXQ1 expression in various solid tumors generated with GeneChip® Human Genome U133 Plus 2.0. A = adenoma, T = tumor carcinoma, N = normal, B = basal, NB = non-basal, D = ductal, L = lobular. Fold changes and rank orders in CRC were corroborated by data mining of TCGA dataset (http://cancergenome.nih.gov) and others in Oncomine (www.oncomine.org).

**Figure 3:**
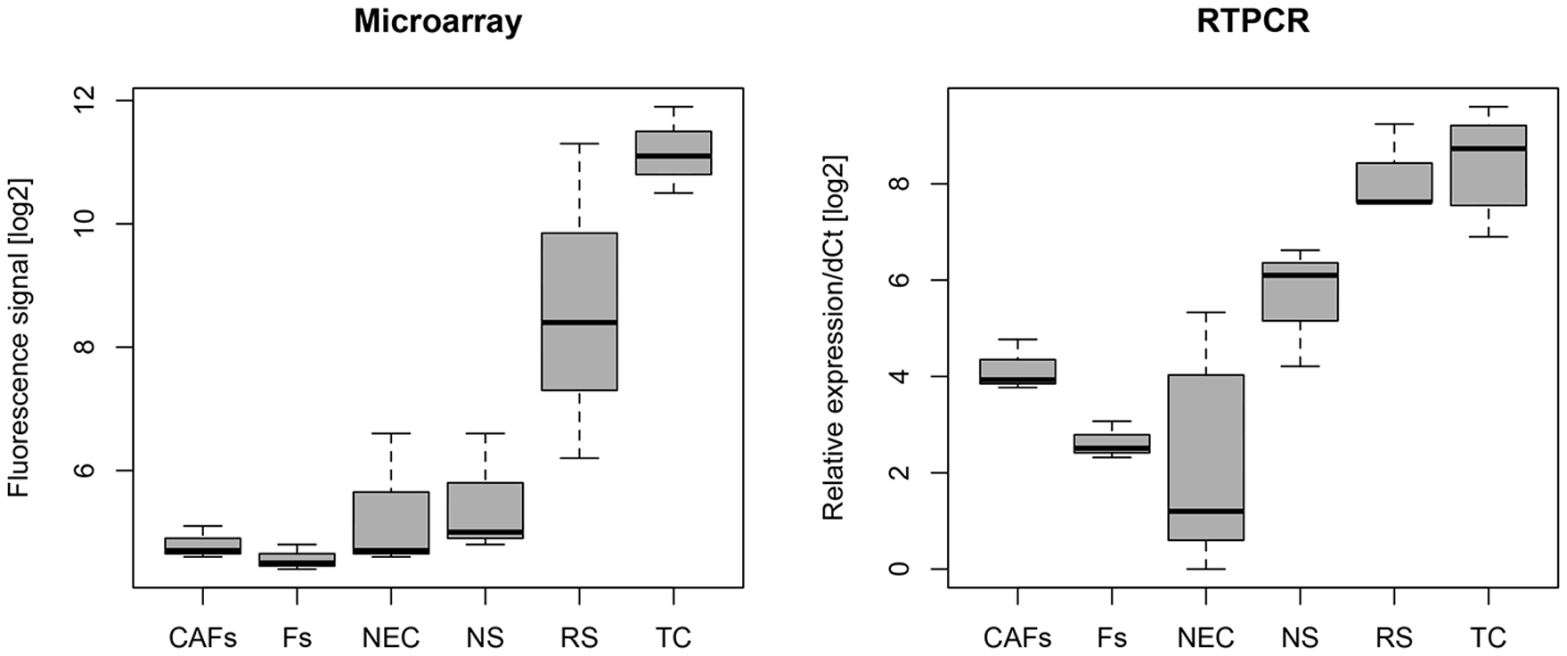
FOXQ1 is expressed in tumor cells and reactive stroma. Boxplot of FOXQ1 mRNA levels in laser dissected human CRC samples measured by microarray n = 3 and qRT-PCR n = 3–5. TC = tumor cells, NEC = normal epithelial cells, NS = normal stroma, i.e. stromal tissue surrounding normal colonocytes, RS = reactive stroma, i.e. stromal tissue with increased infiltration of inflammatory cells surrounding tumor cells, F = primary cells cultures of fibroblasts and CAF = cancer-associated fibroblasts.

Next, we wanted to elucidate the localization of FOXQ1 expressing cells within the primary tumor (details see [21]). Microarray and qRT-PCR analysis of laser-dissected CRC biopsies showed that FOXQ1 was significantly overexpressed in tumor epithelium and reactive stroma tissue (Figure 3). To exclude that FOXQ1 over-expression in CRC samples was not due to increased inflammation we examined FOXQ1 mRNA levels in inflamed colon tissue. FOXQ1 was only very weakly induced in inflamed colon tissue as compared to normal tissue (i.e. 1.2 to 3.3. fold induction as measured in data sets GSE11223 and GSE9452, respectively).

### Association of signalling pathways with FOXQ1

As FOXQ1 was induced in CRC we wanted to test if it was associated with Wnt signalling and/or proliferative activity in CRC. Correlation studies (i.e. PGC) using expO data of CRC samples revealed a strong positive correlation of FOXQ1 expression with direct Wnt targets (AXIN2, APCDD1), epithelial marker (CDH1), intestinal stem cell marker (LGR5) and proliferation-related genes (MKI69, TPX2, AURKA), yet a negative correlation was observed with EMT-markers (VIM, SNAI2, ZEB2, CDH2) (Table S2). GSEA confirmed a significant association between FOXQ1 and direct Wnt targets and gene sets of the MSigDB related to Wnt activation in CRC (Table 2).

**Table 2:** Correlation of FOXQ1 with the Wnt pathway.

Tissue	Direct WNT Targets	SIZE	ES	NOM p-val	FDR q-val
ALL	WNT	24	0.793	0.000	0.018
Breast	WNT	24	0.756	0.000	0.002
Colon	WNT	24	0.723	0.000	0.000
Lung	WNT	24	0.546	0.002	0.008
Pancreas	WNT	24	0.535	0.006	0.022
Prostate	WNT	24	0.427	0.086	0.068

Tissue	MSigDB Gene Sets	SIZE	ES	NOM p-val	FDR q-val
ALL	LIN_WNT_UP	49	0.702	0.001	0.397
ALL	WNT_TARGETS	22	0.738	0.002	0.120
ALL	WNT_SIGNALING	59	0.533	0.008	0.036
ALL	WNTPATHWAY	23	0.616	0.000	0.030
ALL	KENNY_CTNNB1_TARGETS_UP	45	0.481	0.008	0.031
ALL	ST_WNT_BETA_CATENIN_PATHWAY	31	0.508	0.010	0.034
ALL	CTNNB1_ONCOGENIC_SIGNATURE	67	0.728	0.000	0.077
Breast	WNT_TARGETS	22	0.613	0.000	0.263
Breast	CTNNB1_ONCOGENIC_SIGNATURE	67	0.534	0.000	0.430
Colon	LIN_WNT_UP	49	0.588	0.000	0.006
Colon	WNT_TARGETS	22	0.676	0.002	0.009
Colon	WNT_SIGNALING	59	0.471	0.003	0.022
Colon	HSA04310_WNT_SIGNALING_PATHWAY	145	0.389	0.003	0.037
Colon	CTNNB1_ONCOGENIC_SIGNATURE	67	0.532	0.000	0.009
Lung	LIN_WNT_UP	49	0.606	0.003	0.020
Lung	KENNY_CTNNB1_TARGETS_UP	45	0.560	0.002	0.015
Lung	KENNY_CTNNB1_TARGETS_DN	39	0.504	0.003	0.013
Lung	WNT_SIGNALING	59	0.455	0.005	0.013
Lung	WNT_TARGETS	22	0.572	0.005	0.013
Lung	CTNNB1_ONCOGENIC_SIGNATURE	67	0.614	0.000	0.021
Pancreas	WNT_TARGETS	22	0.585	0.002	0.038
Pancreas	LIN_WNT_UP	49	0.459	0.000	0.042
Pancreas	CTNNB1_ONCOGENIC_SIGNATURE	67	0.658	0.002	0.193
Pancreas	LIN_WNT_UP	49	0.480	0.007	0.053
Pancreas	KENNY_CTNNB1_TARGETS_DN	39	0.492	0.009	0.051
Pancreas	CTNNB1_ONCOGENIC_SIGNATURE	67	0.686	0.000	0.035

Gene set enrichment analysis of FOXQ1 with a selection of gene set related to Wnt signalling. GSEA preformed in various solid tumors showed a significant enrichment when using direct Wnt targets and gene sets related to Wnt activation. SIZE = size of tested gene set, ES = enrichment score, NOM p-val = Nominal p-value, FDR q-val = false discovery rate q-value.

In order to validate the *in silico* analysis, we knocked down FOXQ1, using shRNA interference, and analysed the expression of genes which strongly correlated with FOXQ1 expression in colon cancer biopsy samples and EMT-related genes, which did not correlate with FOXQ1. Knock down of FOXQ1 showed no significant reduction on protein level (data not shown) but yielded a 1.9 and 2.8 fold reduction of FOXQ1 mRNA levels in SW480 CRC cells (Table S3). Despite the weak knock down efficiency the expression levels were reduced in 14 out of 17 genes whose expression was highly correlated with FOXQ1. However the expression of EMT related genes were not altered (Table S3) thus corroborating the *in silico* data.

### FOXQ1 expression is regulated by the Wnt pathway

**Figure 4:**
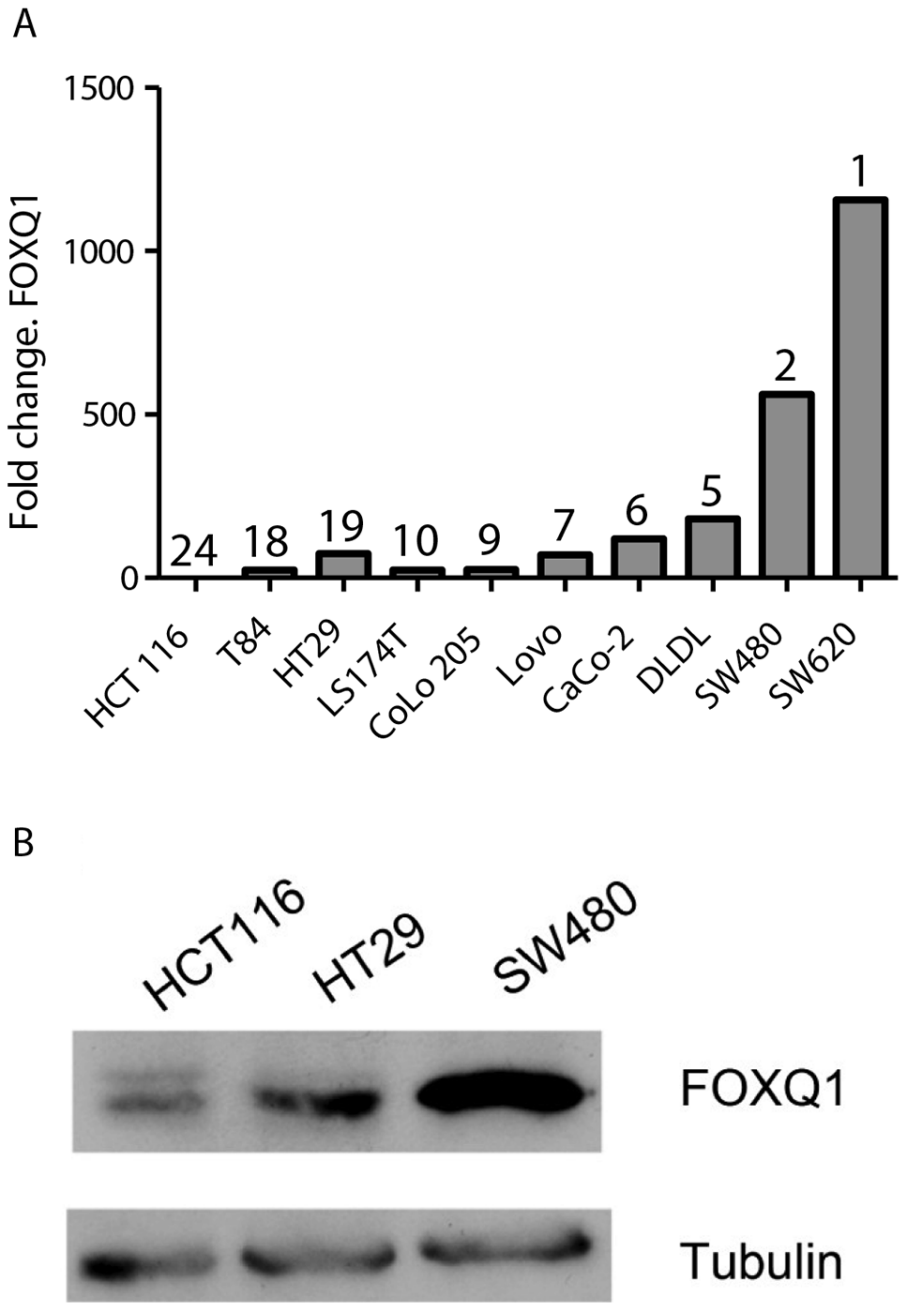
FOXQ1 expression correlates with overall expression strength of direct Wnt target in CRC cell lines. (**A**) FOXQ1 mRNA levels were measured by qRT-PCR in a panel of CRC cell lines. Numbers indicate ranks in terms of Wnt signalling strength according to Christensen et al. [21], with rank 1 having the strongest mean Wnt signal strength. (**B**) Western blot of selected CRC cell lines showing the expression level of FOXQ1. Tubulin was used as loading control.

**Figure 5:**
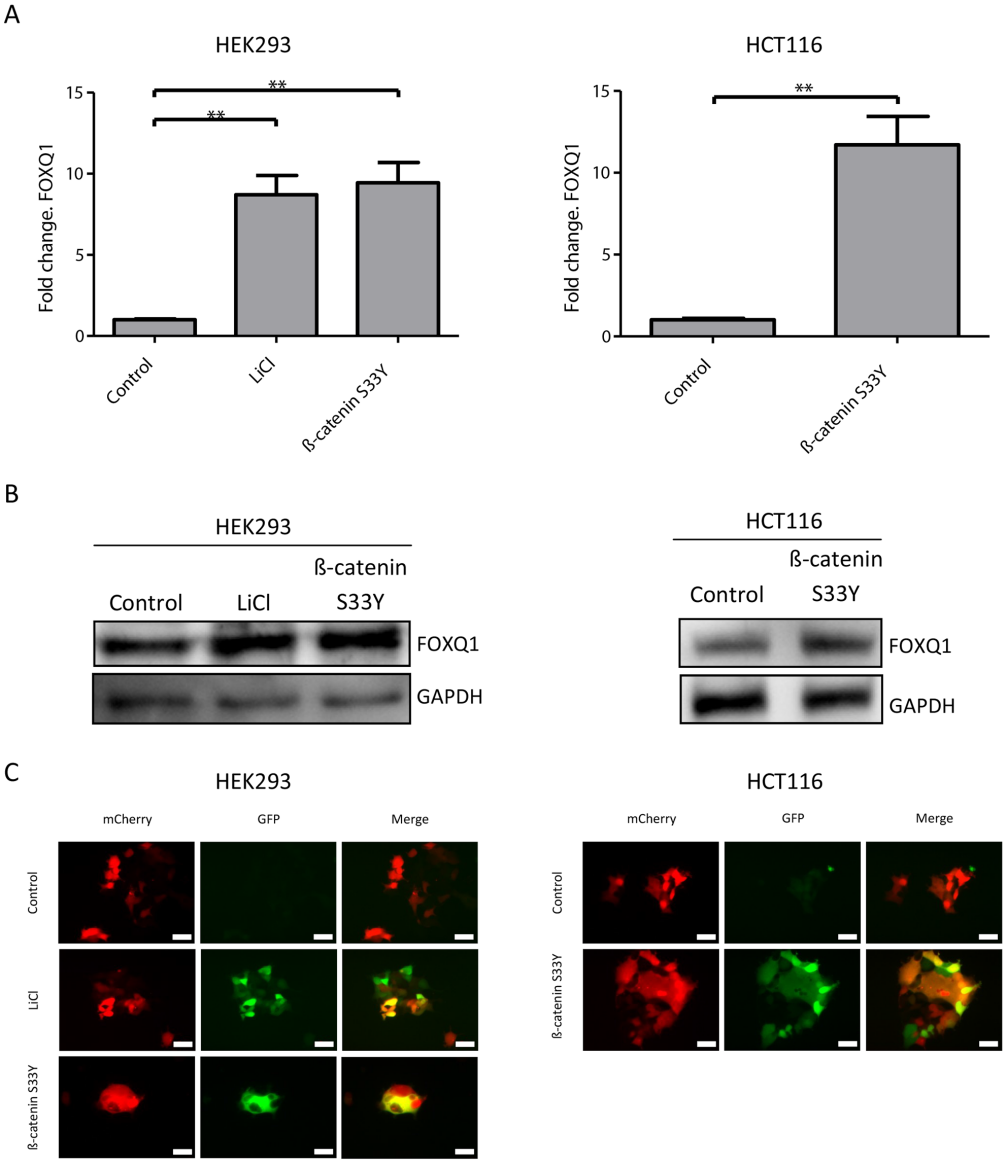
Activation of the Wnt pathway leads to increased FOXQ1 expression. (**A**) qRT-PCR analysis of FOXQ1 expression in HEK293 and HCT116 cell lines after Wnt activation. Cell were treated with 20 mM LiCl for 24 hours or transfected with a constitutively active form of ß-catenin (S33Y). Data are mean ± SD n = 3, **P<0.01 using Mann-Whitney test. (**B**) Western blot of FOXQ1 in HEK293 and HCT116 after Wnt activation as described above. GAPDH was used a loading control. (**C**) HEK293 and HCT116 cells stably expressing the Wnt sensitive reporter 7TGC [25] were treated as described above and imaged. GFP (green) is under the control of a Wnt sensitive promoter and mCherry (red) is constitutively expressed to identify infected cells, white bar indicates 20 µm.

Next, we wanted to test if the expression of FOXQ1 could be linked with Wnt activity of CRC cell lines. Hence, the expression of FOXQ1 was measured in a wide panel of CRC cell lines with different Wnt activity [21], [31]. A correlation was observed between the relative FOXQ1 mRNA and protein expression with the ranking of cell lines according to their Wnt signature expression (Figure 4). Additional evidence for a possible regulation of FOXQ1 through the Wnt pathway was obtained in Caco-2 cells, where FOXQ1 upregulation was observed upon loss of CDX2 expression (Table 3). CDX2 has been shown to inhibit Wnt signalling in Caco-2 cells [32]. Both HEK293 and the CRC cell line HCT116 have an intact Wnt pathway and overall low Wnt signature strength. Activation of the canonical Wnt pathway with a small molecular GSK-3 inhibitor (LiCl) or by introducing a constitutively active form of ß-catenin (S33Y) led to increases in FOXQ1 mRNA and protein level (Figure 5A–5B) [22]–[24]. The Wnt sensitive reporter 7TGC [25] was used to show that LiCl and ß-catenin (S33Y) treatment activated the canonical Wnt pathway (Figure 5C).

**Table 3:** Regulation of FOXQ1 expression upon loss of CDX2 expression.

Sample Name	CDX2	AXIN2	FOXQ1
sh1_5days	−1.2	2.5	3.7
sh2_5days	−1.2	2.0	2.5
sh3_5days	−1.4	2.1	2.2
sh1_3weeks	−1.8	2.3	3.2
sh2_3weeks	−1.6	1.3	2.4
sh3_3weeks	−1.5	1.8	2.3

Relative expression levels (fold changes) of CDX2, FOXQ1 and AXIN2 in shCDX2 Caco-2 cells grown for 5 days and 3 weeks as compared to control transfected cells, measured by qRT-PCR.

### ß-catenin directly regulates transcription of FOXQ1

**Figure 6:**
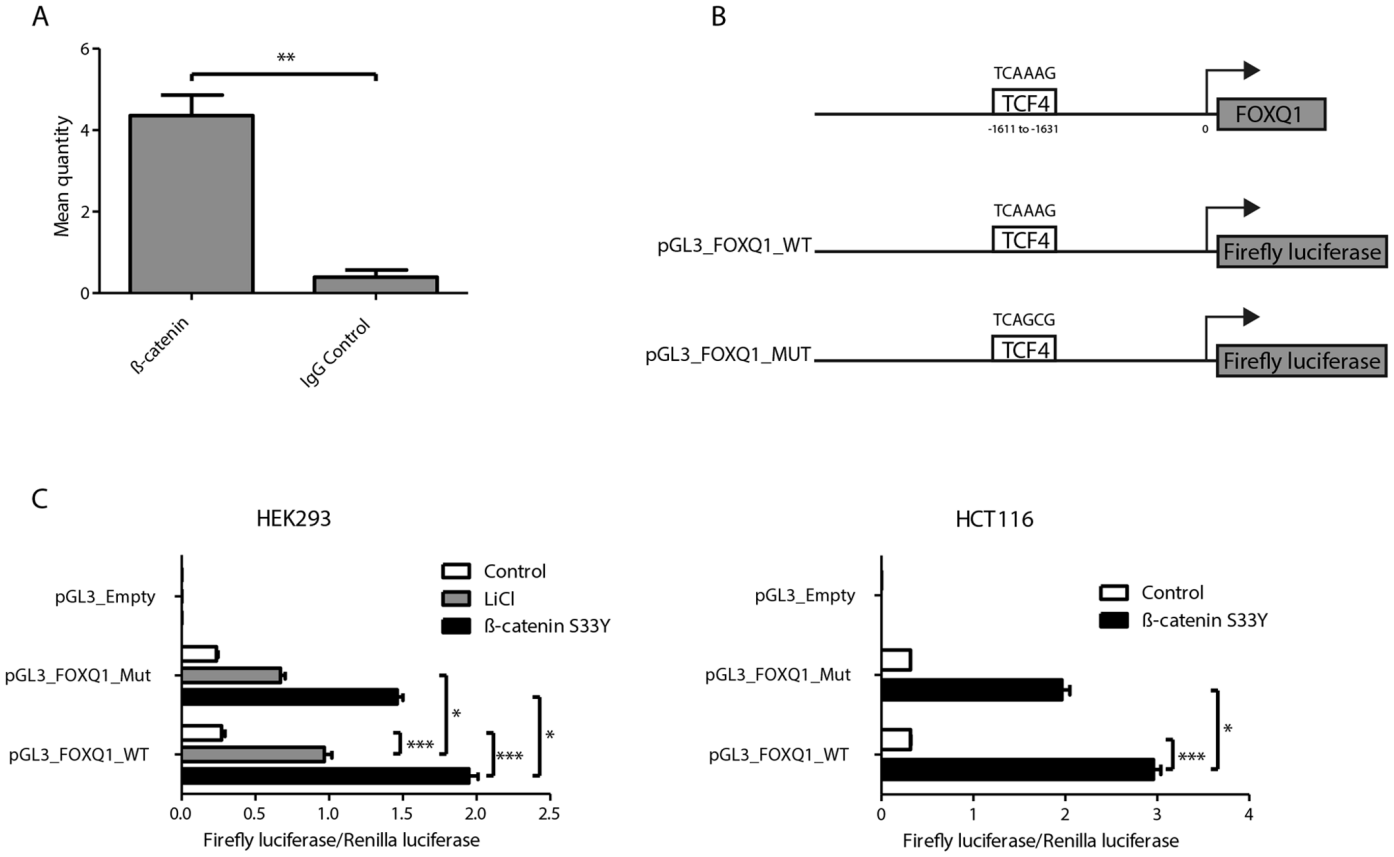
ß-catenin binds to the promoter region of FOXQ1 and increase transcription. (**A**) ChIP assay was performed on SW480 cells with antibodies against ß-catenin and IgG (control). Input and immunoprecipitated DNA was measured by qRT-PCR using primers amplifying the promoter region of FOXQ1 and the −100/0 promoter region of GAPDH. Data are mean ± SD n = 3, **P<0.01 using Mann-Whitney test. (**B**) Graphical depiction of the promoter region of FOXQ1, and the generated promoter reporter vectors. (**C**) Luciferase assay showing the effect of Wnt activation in HEK293 and HCT116 cells (as described in figure 5) transfected with the wild type promoter region of FOXQ1 (pGL3_FOXQ1_WT), a mutated TCF4 binding site (pLG3_FOXQ1_Mut) or an empty vector (pGL3_Emtpy). Data are mean ± SD n = 4–7, *P<0.05, ***P<0.001 by the Kruskal-Wallis Conover test.

In order to investigate whether ß-catenin directly drives transcription of FOXQ1, a 2.5 kb region upstream of the transcription start site (TSS) of the FOXQ1 gene was analysed for the existence of TCF4 binding sites using Transcription Element Search System (TESS). The presence of a highly conserved TCF4 binding site was found -1.634 kb upstream of the TSS with a core TCF-4 binding motif (TCAAAG) [33], [34]. To demonstrate that ß-catenin was directly bound to the promoter region of FOXQ1 we conducted ChIP experiments in the CRC cell line SW480 which have a strong Wnt signalling activity [31]. An anti-ß-catenin antibody specifically enriched fragments of the FOXQ1 promoter region that contained the putative TCF-4 binding site compared to IgG antibody control (Figure 6A). Luciferase reporter assays showed that activation of the Wnt pathway increased transcriptional activity of the wildtype FOXQ1 promoter. Mutation of the TCF-4 binding site TCAAAG to TCAGCG (Figure 6B) resulted in a significantly decreased signal upon Wnt activation although not a complete abrogation (Figure 6C), thus showing that the Wnt pathway regulates FOXQ1 expression in part via the identified TCF-4 binding site.

### FOXQ1 as marker for Wnt activation in solid tumors of different tissue origins

**Figure 7:**
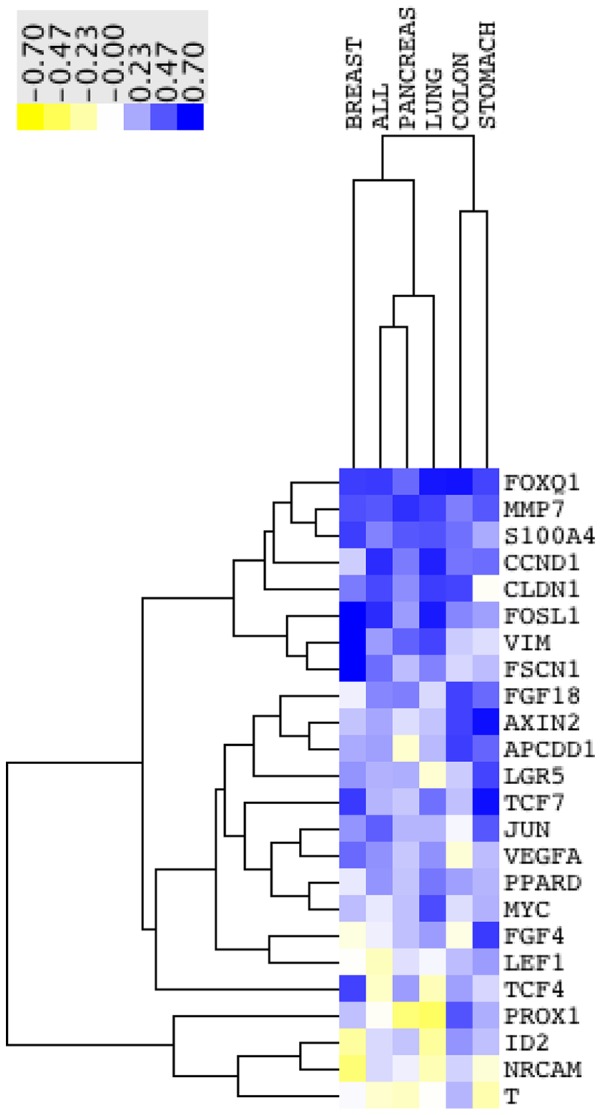
FOXQ1 expression correlates with the average Wnt signature strength in a wide panel of cell lines. Heatmap showing the correlation coefficients between single genes and the mean expression of all 24 direct Wnt targets in various cancer cell lines (data set GSE36133). Colon n = 57, breast n = 58, lung = 174, pancreas n = 44, stomach n = 38, all n = 967.

Activation of the Wnt pathway is a well-defined hallmark in CRC, however, studies suggest its importance in tumor progression of other epithelial tissues such breast, lung, prostate and pancreas [8], [10], [11], [35]–[37]. Therefore, we wanted to investigate the potential of FOXQ1 as a marker to assess Wnt activation in other tumors. PGC using expO data revealed that breast, prostate and colon tumors had a strong association between FOXQ1 and Wnt related genes (t values>3 for AXIN2 and APCCD1) (Table S2). GSEA of direct Wnt targets and genes related to Wnt activation showed a strong positive enrichment in colon, breast, lung and pancreas (Table 2). Moreover, we wanted to elucidate to which extent FOXQ1 could serve as marker to assess Wnt activation in *in vitro* models. Out of the direct 24 direct Wnt targets studied, expression levels of FOXQ1, FOSL1 and CCND1 correlated best with the mean expression of all direct Wnt targets across a panel of 967 cell lines of different tissues (Figure 7). Focusing on carcinoma cell lines derived from colon, breast, lung, pancreas and stomach FOXQ1 ranked best in terms of correlation with the mean expression of all Wnt targets suggesting it to be a suitable and sensitive marker for Wnt activation in cell lines (Figure 7).

## Discussion

In this study, using microarray data from cancer cell lines and patient samples, and molecular biological techniques we examined the relationship between FOXQ1 and the Wnt pathway. FOXQ1 expression was highly correlated with the activity of the Wnt pathway in primary tumors and cell lines of epithelial origin. FOXQ1 mRNA and protein levels could be increased by activating of the canonical Wnt pathway via LiCl or by introduction of a constitutively active form of ß-catenin (S33Y). ß-catenin was bound to the promoter region of FOXQ1 at a highly conserved TCF-4 binding site. The wildtype promoter region was transcriptional active when the Wnt pathway was activated. Mutation of the TCF-4 binding decreased the transcriptional activity of the promoter upon Wnt activation.

Recent reports have shown that FOXQ1 was upregulated in CRC, metastatic breast cancer cell lines and FOXQ1 expression level in lung and breast cancer patients was highly correlated with EMT markers [17]–[19], [38] However, in CRC, FOXQ1 expression was independent of grade, location and metastasis (Figure 2, Figure S1), despite being among the most upregulated genes in CRC (Table 1). FOXQ1 overexpression did not influence overall or recurrence free survival in CRC patients; this is in contrast to data from breast and lung cancer patients where FOXQ1 was associated with worse survival [18], [38]. Also, in contrast to findings in breast cancer we could not observe an association of FOXQ1 with EMT features in CRC (Table S2, Table S3 and Table S4). Interestingly, no association was found between Wnt signalling and EMT features in CRC (data not shown). Contribution of Wnt activation to tumorigenesis may vary depending on the tissue of origin. Wnt activation is an early and frequent event in CRC owing to mutations of various genes in the Wnt signalling cascade [7]. This results in an initial increase of basal Wnt activity in the tumors. In contrast, increased Wnt signalling in breast and lung tumors are most likely due to external cues rather than mutation [39], [40]. Also, Wnt activation is a later event that is associated with tumor progression and a more invasive phenotype [8], [10], [36].

The Wnt pathway is emerging as an important regulator of various cancer hallmarks and as a prognostic tool. ß-catenin staining is the standard for analysing Wnt activation but mRNA analysis techniques such as qRT-PCR and microarray are important tools in basic and clinical research. It is important to have reliable mRNA markers for Wnt activation that can be analysed in different tissues. Compared to 24 previously known Wnt targets FOXQ1 expression level was one of the best markers for assessing Wnt activity in a panel of 967 cell lines derived from different tissues. Also in primary tumors FOXQ1 correlated with signatures related to the Wnt pathway. This data indicates that FOXQ1 could potentially be used as a Wnt marker in multiple tissues.

In summary, we showed for the first time the molecular events underpinning the increased expression of FOXQ1 in cancers, and show the usefulness of FOXQ1 as a marker for Wnt activity in cancer cell lines.

## Supporting Information

Figure S1 **FOXQ1 expression is not influenced by stage, grade and localization of the tumor.** Boxplots of FOXQ1 mRNA expression levels (i.e. normalized, log2 transformed fluorescence signals) in the GSE5206 data set. Stages: stage A, B, C and D; Grades: well = well differentiated, mod = moderately differentiated and poorly = poorly differentiated; Localization: S = sigmoid colon, A = ascending colon, CE = cecum, R = rectum, T = transverse colon and D = descending. Metastasis: non-metastatic = no metastatic site observed and metastatic = metastatic site observed.
(PDF)

Figure S2 **FOXQ1 is highest expressed in human CRC.** Boxplots of FOXQ1 mRNA (normalized fluorescence signal levels, log2 transformed) in human biopsy samples of various tumors (expO data set, GSE2109). BR = breast, CO = colon, KI = kidney, OV = ovary, UT = uterus, LU = lung, PR = prostate, EN = endometrium, OM = omentum, LI = liver.
(PNG)

Figure S3 **FOXQ1 is highest expressed in cell lines derived from CRC.** Boxplots of FOXQ1 mRNA expression level (normalized fluorescence signal levels, log2 transformed) in the NCI60 cell line panel. BR = breast, CNS = central nervous system, CO = colon, LC = lung, LE = leukocytes, ME = melanoma, OV = ovarian, PR = prostrate, RE = rectum
(PDF)

Figure S4 **Expression of Wnt targets and FOXQ1 in a wide panel of cancer derived cell lines.** Boxplots of FOXQ1 mRNA expression level (normalized fluorescence signal levels, log2 transformed), and direct Wnt targets CCND1, FOSL1 and AXIN2 [log2 transformed] in 967 cancer cell lines derived from different tissues (GSE36133).
(PDF)

Table S1 **Primers sequences for Real-Time PCR.**
(DOCX)

Table S2 **Strong association of FOXQ1 expression with genes involved in Wnt signaling and proliferation.** T-statistics based on Prototype-based Gene Coexpression analysis (expO data set (GSE2109)) with respect to the correlation of FOXQ1 with various known direct Wnt targets (AXIN2, APCDD1), markers for proliferation (MKI67, TPX2, AURKA), mesenchymal cells/EMT (VIM, SNAI1, ZEB2, CDH2), differentiated epithelial cells (CDH1) and intestinal stem cells (LGR5).
(DOCX)

Table S3 **Confirmation of in silico analysis.** FOXQ1 was knocked down in SW480 cells using two different shRNA constructs (KD1 and KD2). The relative expression levels (fold changes) of genes strongly correlating with FOXQ1 expression in human colon cancer biopsy samples and or genes associated involved in EMT were measured using qRT-PCR. Data is shown as fold changes compared to wild type SW480. T-statistics based on Prototype-based Gene Coexpression analysis of FOXQ1 using the expO data set (GSE2109).
(DOCX)

Table S4 **Significant enrichment of direct EMT-related genes targets in tumors.** Gene set enrichment analysis with an EMT-related gene set (see [29]).
(DOCX)
