# Supplementary figures and images for: FOXQ1, a Novel Target of the Wnt Pathway and a New Marker for Activation of Wnt Signaling in Solid Tumors
Source: PLoS One. 2013 Mar 26;8(3):e60051. doi: 10.1371/journal.pone.0060051 (PMC3608605; doi:10.1371/journal.pone.0060051)

**Stage**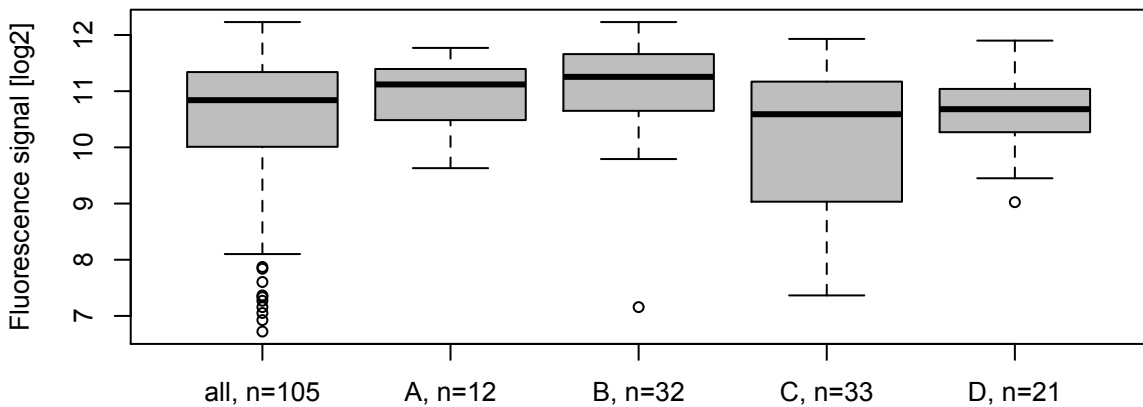**Grade**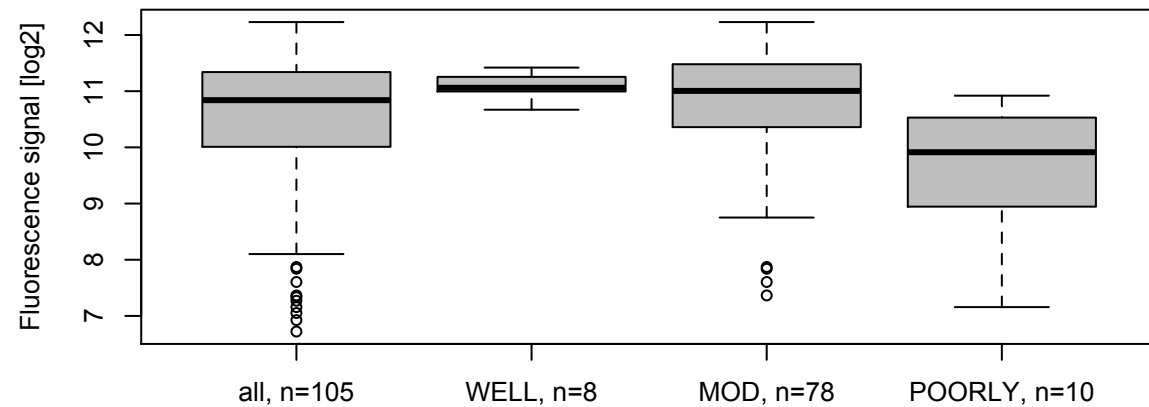**Localization**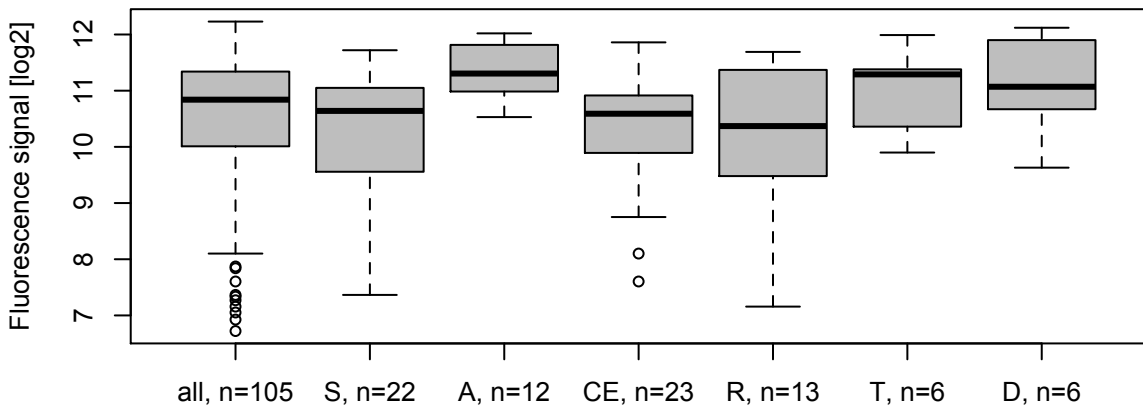**Metastasis**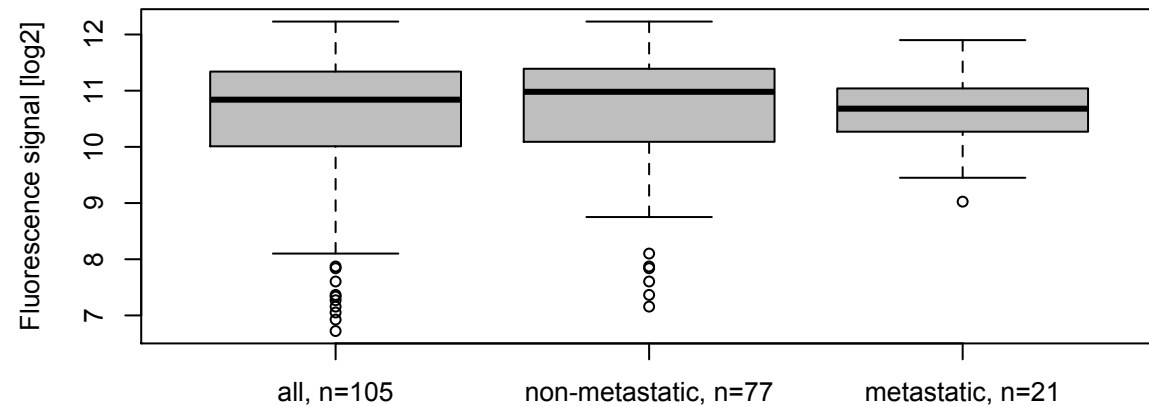

Supplement: Figure S1 — FOXQ1 expression is not influenced by stage, grade and localization of the tumor. Boxplots of FOXQ1 mRNA expression levels (i.e. normalized, log2 transformed fluorescence signals) in the GSE5206 data set. Stages: stage A, B, C and D; Grades: well = well differentiated, mod = moderately differentiated and poorly = poorly differentiated; Localization: S = sigmoid colon, A = ascending colon, CE = cecum, R = rectum, T = transverse colon and D = descending. Metastasis: non-metastatic = no metastatic site observed and metastatic = metastatic site observed. (PDF) [file pone.0060051.s001.pdf]

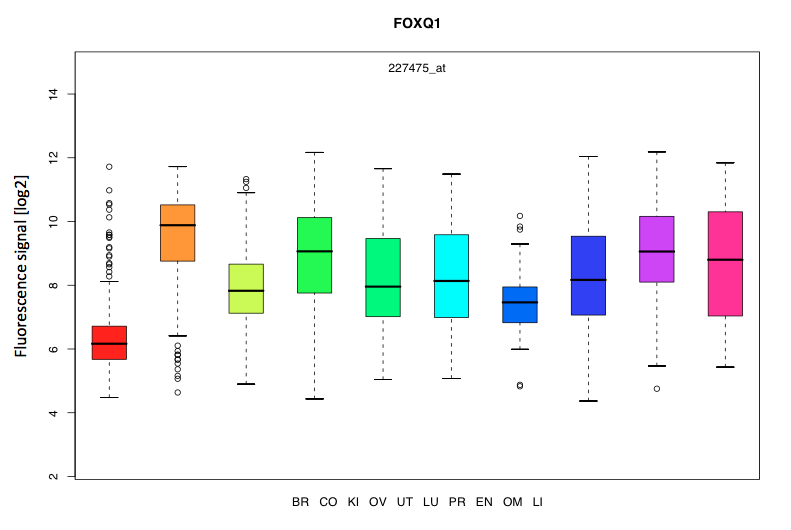

Supplement: Figure S2 — FOXQ1 is highest expressed in human CRC. Boxplots of FOXQ1 mRNA (normalized fluorescence signal levels, log2 transformed) in human biopsy samples of various tumors (expO data set, GSE2109). BR = breast, CO = colon, KI = kidney, OV = ovary, UT = uterus, LU = lung, PR = prostate, EN = endometrium, OM = omentum, LI = liver. (PNG) [file pone.0060051.s002.png]

NCI60 panel

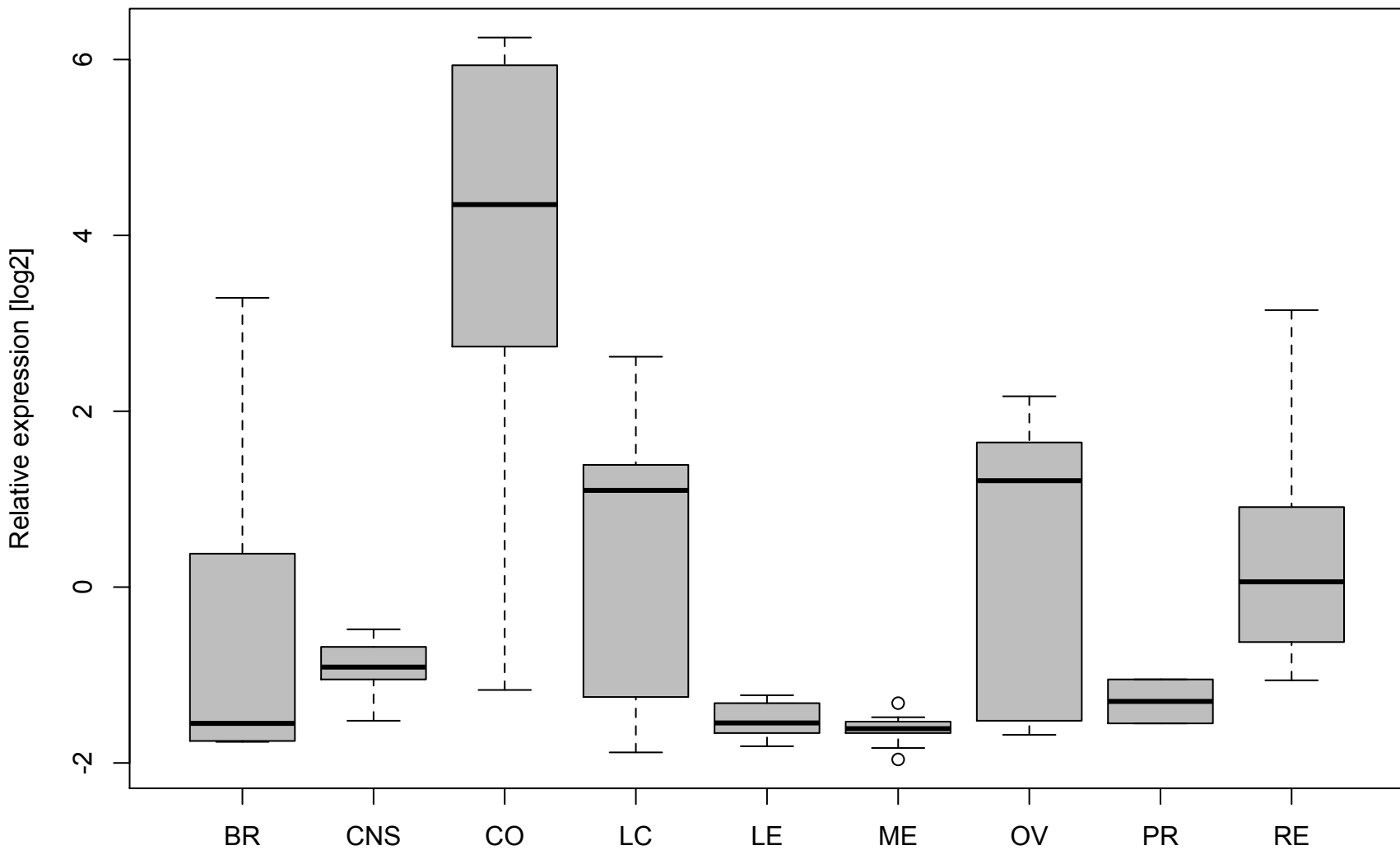

Supplement: Figure S3 — FOXQ1 is highest expressed in cell lines derived from CRC. Boxplots of FOXQ1 mRNA expression level (normalized fluorescence signal levels, log2 transformed) in the NCI60 cell line panel. BR = breast, CNS = central nervous system, CO = colon, LC = lung, LE = leukocytes, ME = melanoma, OV = ovarian, PR = prostrate, RE = rectum (PDF) [file pone.0060051.s003.pdf]

**ALL,n=967**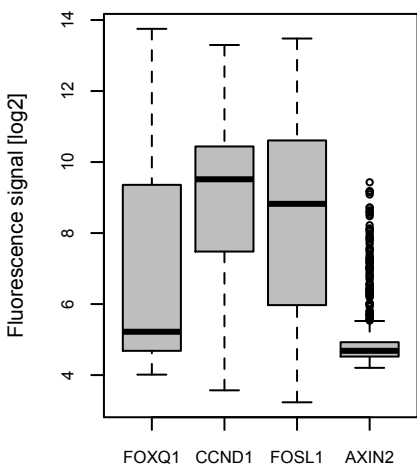**COLON,n=57**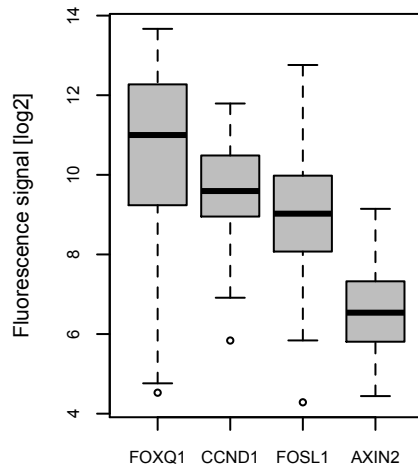**LUNG,n=174**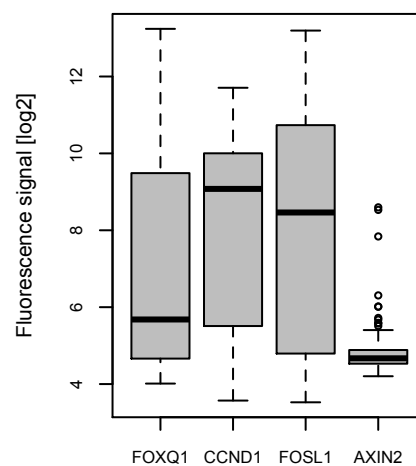**BREAST,n=58**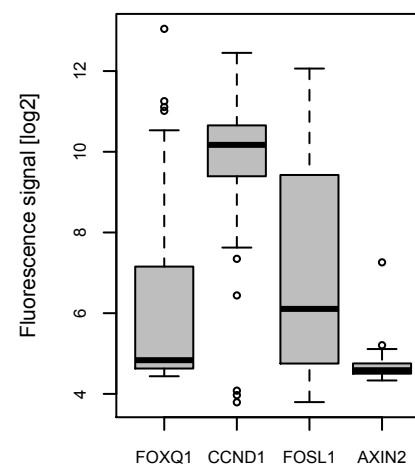**PROSTATE,n=7**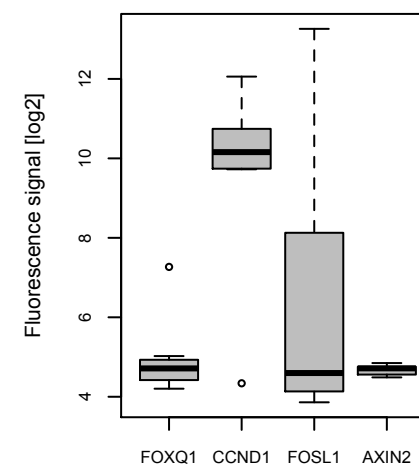**PANCREAS,n=44**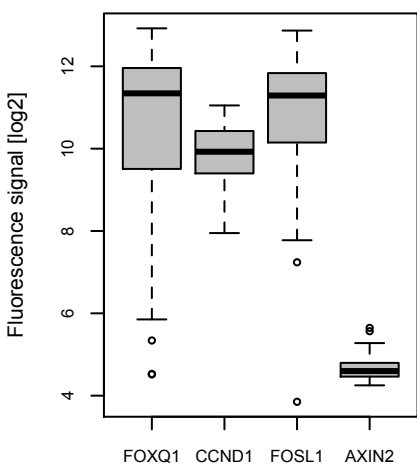**KIDNEY,n=22**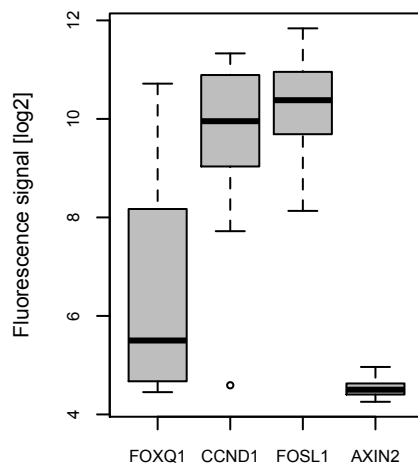**SKIN,n=60**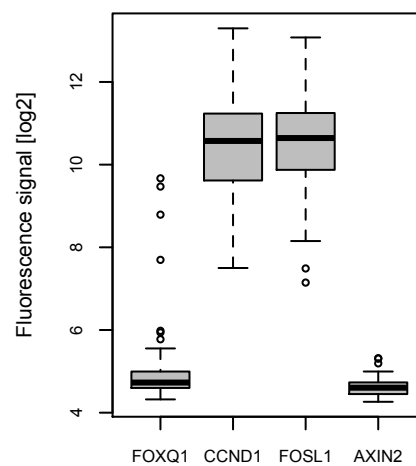**BONE,n=25**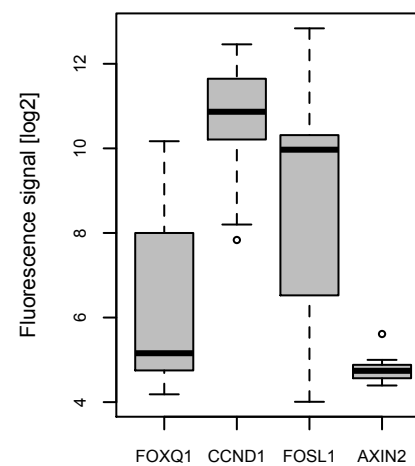**BLOOD,n=178**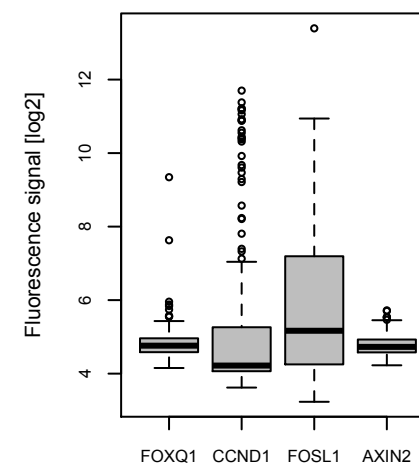

Supplement: Figure S4 — Expression of Wnt targets and FOXQ1 in a wide panel of cancer derived cell lines. Boxplots of FOXQ1 mRNA expression level (normalized fluorescence signal levels, log2 transformed), and direct Wnt targets CCND1, FOSL1 and AXIN2 [log2 transformed] in 967 cancer cell lines derived from different tissues (GSE36133). (PDF) [file pone.0060051.s004.pdf]
