# Supplementary material for: FOXQ1, a Novel Target of the Wnt Pathway and a New Marker for Activation of Wnt Signaling in Solid Tumors
Source: PLoS One. 2013 Mar 26;8(3):e60051. doi: 10.1371/journal.pone.0060051 (PMC3608605; doi:10.1371/journal.pone.0060051)
Supplement: Table S1 — Primers sequences for Real-Time PCR. (DOCX) [file pone.0060051.s005.docx]

Table S1: **Primers sequences for Real-Time PCR.**

| **Primer Name** | **Sequence** |
| --- | --- |
| Forw_FOXQ1 | CTTCCCTCCCCCCTAAGTACAT |
| Rev_FOXQ1 | ATGCCACATACGTACACGGATG |
| Forw_ChIP_FOXQ1 | ATGGCTTCAAAACTCCCTGA |
| Rev_ChIP_FOXQ1 | GACGGAGCCGCTAGTAGGT |
| Forw_ChIP_GAPDH | TCCTCCTGTTTCATCCAAGC |
| Rev_ChIP_GAPDH | TAGTAGCCGGGCCCTACTTT |
| Forw_HMBS | GAGTGATTCGCGTGGGTACC |
| Rev_HMBS | CACACTGTCCGTCTGTATGCG |
| Forw_H3 | AGACTGCCCGCAAATCGAC |
| Rev_H3 | CTTGCGAGCGGCTTTTGTA |
| Forw_Promoter_Mut | GTAATAAAATAAACATCAGCGGTGAGCTCCCACCTACTAGCGGC |
| Rev_Promoter_Mut | GCCGCTAGTAGGTGGGAGCTCACCGCTGATGTTTATTTTATTAC |
| Forw_Promoter_Cloning | GCTAGCCCACGGCCTGCCATGCCATCT |
| Rev_Promoter_Cloning | CCCAAAGTCTCAACGTCGAACCGGAGCTC |
