# Supplementary material for: FOXQ1, a Novel Target of the Wnt Pathway and a New Marker for Activation of Wnt Signaling in Solid Tumors
Source: PLoS One. 2013 Mar 26;8(3):e60051. doi: 10.1371/journal.pone.0060051 (PMC3608605; doi:10.1371/journal.pone.0060051)
Supplement: Table S2 — Strong association of FOXQ1 expression with genes involved in Wnt signaling and proliferation. T-statistics based on Prototype-based Gene Coexpression analysis (expO data set (GSE2109)) with respect to the correlation of FOXQ1 with various known direct Wnt targets (AXIN2, APCDD1), markers for proliferation (MKI67, TPX2, AURKA), mesenchymal cells/EMT (VIM, SNAI1, ZEB2, CDH2), differentiated epithelial cells (CDH1) and intestinal stem cells (LGR5). (DOCX) [file pone.0060051.s006.docx]

Table S2: **Strong association of FOXQ1 expression with genes involved in Wnt signaling and proliferation.** T-statistics based on Prototype-based Gene Coexpression analysis (expO data set (GSE2109)) with respect to the correlation of FOXQ1 with various known direct Wnt targets (AXIN2, APCDD1), markers for proliferation (MKI67, TPX2, AURKA), mesenchymal cells/EMT (VIM, SNAI1, ZEB2, CDH2), differentiated epithelial cells (CDH1) and intestinal stem cells (LGR5).

| **Gene** | **ALL** | **Colon** | **Breast** | **Lung** | **Pancreas** | **Prostate** |
| --- | --- | --- | --- | --- | --- | --- |
| AXIN2 | 4.05 | 10.59 | 3.73 | 2.19 | 2.45 | 3.11 |
| APCDD1 | 3.43 | 5.92 | 5.59 | 1.29 | 2.97 | 2.26 |
| LGR5 | 2.09 | 9.46 | 5.63 | 1.6 | 2.33 | 0.68 |
| MKI67 | 0.64 | 6.44 | 1.87 | 0.11 | 2.11 | -2.44 |
| TPX2 | 0.68 | 8.98 | 0.82 | -0.58 | 1.85 | -1.68 |
| AURKA | 0.7 | 8.1 | 0.36 | -0.43 | 2.34 | -2.02 |
| CDH1 | 3.48 | 7.24 | -1.3 | 2.83 | 3.04 | 2.32 |
| VIM | -0.59 | -4.4 | 0.54 | 1.19 | -0.68 | -0.35 |
| SNAI2 | -0.44 | -1.94 | -0.1 | 0.36 | -0.09 | 1.59 |
| ZEB2 | -1.44 | -5.16 | -2.48 | 1.34 | -0.83 | 1.03 |
| CDH2 | 0.34 | -2.68 | 5.35 | -3.47 | 1.32 | 1.6 |
