# Supplementary material for: FOXQ1, a Novel Target of the Wnt Pathway and a New Marker for Activation of Wnt Signaling in Solid Tumors
Source: PLoS One. 2013 Mar 26;8(3):e60051. doi: 10.1371/journal.pone.0060051 (PMC3608605; doi:10.1371/journal.pone.0060051)
Supplement: Table S3 — Confirmation of in silico analysis. FOXQ1 was knocked down in SW480 cells using two different shRNA constructs (KD1 and KD2). The relative expression levels (fold changes) of genes strongly correlating with FOXQ1 expression in human colon cancer biopsy samples and or genes associated involved in EMT were measured using qRT-PCR. Data is shown as fold changes compared to wild type SW480. T-statistics based on Prototype-based Gene Coexpression analysis of FOXQ1 using the expO data set (GSE2109). (DOCX) [file pone.0060051.s007.docx]

Table S3: **Confirmation of in silico analysis.** FOXQ1 was knocked down in SW480 cells using two different shRNA constructs (KD1 and KD2). The relative expression levels (fold changes) of genes strongly correlating with FOXQ1 expression in human colon cancer biopsy samples and or genes associated involved in EMT were measured using qRT-PCR. Data is shown as fold changes compared to wild type SW480. T-statistics based on Prototype-based Gene Coexpression analysis of FOXQ1 using the expO data set (GSE2109).

| **Target Name** | **KD1** | **KD2** | **t-statistics** |
| --- | --- | --- | --- |
| FOXQ1 | -1.9 | -2.8 | Inf |
| Genes correlating with FOXQ1 (high t values) | | | |
| PERP | -1.0 | -1.1 | 12.5 |
| RNF43 | -1.7 | -1.6 | 11.4 |
| FERMT1 | -1.8 | -1.5 | 11.2 |
| SLCO4A1 | -1.4 | -1.7 | 10.8 |
| ASCL2 | 1.1 | 1.1 | 10.3 |
| SLC6A6 | -1.5 | -1.8 | 10.2 |
| SLC7A1 | -1.8 | -1.9 | 9.4 |
| MET | -1.0 | -1.3 | 8.9 |
| SLC5A6 | -1.3 | -1.2 | 8.8 |
| MACC1 | -2.0 | -1.9 | 8.3 |
| SLC7A5 | -1.7 | -1.8 | 7.3 |
| SLC7A5 | -1.9 | -1.9 | 7.3 |
| CDH1 | -2.4 | -2.4 | 7.2 |
| MYC | -1.1 | 1.1 | 7.2 |
| CDX2 | -1.8 | -1.3 | 6.7 |
| MKI67 | -1.9 | -1.5 | 6.4 |
| SLC12A2 | -1.3 | -1.1 | 6.4 |
| EMT-related genes | |  |  |
| TGFB1 | -2.1 | -2.6 | -1.8 |
| ZEB1 | -1.0 | 1.4 | -1.9 |
| SNAI2 | -1.3 | -1.6 | -1.9 |
| CDH2 | -1.0 | -1.2 | -2.7 |
| VIM | -1.0 | -1.0 | -4.4 |
