# Supplementary material for: FOXQ1, a Novel Target of the Wnt Pathway and a New Marker for Activation of Wnt Signaling in Solid Tumors
Source: PLoS One. 2013 Mar 26;8(3):e60051. doi: 10.1371/journal.pone.0060051 (PMC3608605; doi:10.1371/journal.pone.0060051)
Supplement: Table S4 — Significant enrichment of direct EMT-related genes targets in tumors. Gene set enrichment analysis with an EMT-related gene set (see [29]). (DOCX) [file pone.0060051.s008.docx]

Table S4: **Significant enrichment of direct EMT-related genes targets in tumors.** Gene set enrichment analysis with an EMT-related gene set (see [29]).

| **Tissue** | **Gene set** | **Size** | **ES** | **NOM p-val** | **FDR q-val** |
| --- | --- | --- | --- | --- | --- |
| BR | EMT | 14 | 0.505 | 0.037 | 0.056 |
| CO | EMT | 14 | -0.356 | 0.462 | 0.521 |
| LU | EMT | 14 | -0.356 | 0.460 | 0.508 |
| PR | EMT | 14 | 0.260 | 0.802 | 0.946 |
